# Secondary myoadenylate deaminase deficiency is not a common feature of inflammatory myopathies: A descriptive study

**DOI:** 10.3389/fmed.2022.1061722

**Published:** 2022-11-23

**Authors:** Michael Wilkinson, Kathy Cash, Bernice Gutschmidt, Sophia Otto, Vidya Limaye

**Affiliations:** ^1^Faculty of Health and Medical Sciences, University of Adelaide, Adelaide, SA, Australia; ^2^Department of Rheumatology, Flinders Medical Centre, Adelaide, SA, Australia; ^3^Department of Rheumatology, Royal Adelaide Hospital, Adelaide, SA, Australia; ^4^Muscle and Nerve Laboratory, Department of Anatomical Pathology, SA Pathology, Adelaide, SA, Australia

**Keywords:** myoadenylate deaminase, myositis, metabolic myopathy, muscle biopsy, autoimmune disease

## Abstract

**Background:**

Myoadenylate deaminase (MAD) deficiency is a form of metabolic myopathy, which generally causes only mild symptoms in the primary inherited form. Inflammatory myopathies are a group of autoimmune diseases which result in skeletal muscle weakness. In addition to inflammatory pathology, it has been speculated that non-inflammatory mechanisms, and possibly secondary MAD-deficiency, may potentially contribute to weakness in these conditions.

**Methods:**

We investigated for an association between these two myopathic processes through two complementary methods. Firstly, muscle biopsy records in South Australia over a 17-year period were retrospectively reviewed for diagnosis of myositis or MAD-deficiency, as well as associated clinical features. Secondly, a prospective arm histochemically tested all incident biopsy specimens over a 12-month period for MAD-deficiency.

**Results:**

In the retrospective arm, 30 MAD-deficient cases were identified (1.3% of all biopsies), with no significant difference observed in overall rates of myositis diagnosis between patients with intact and deficient MAD activity (21.3% vs 26.7%, *P* = 0.47). No cases of MAD-deficiency were detected in the prospective arm, despite 39 cases of myositis being identified over this period.

**Conclusion:**

Secondary MAD deficiency is unlikely to be a major driver of symptoms in inflammatory myopathies.

## Introduction

Myoadenylate Deaminase (MAD) Deficiency is a form of metabolic myopathy which may cause exertional myalgia and reduced exercise tolerance through deficiency in the enzyme Myoadenylate Deaminase (MAD) (1). Inherited cases are primarily caused by nonsense mutations (C34T most commonly) in the AMPD1 gene, exhibiting an autosomal recessive pattern with incomplete penetrance (1, 2). The enzyme’s function is to convert adenosine monophosphate into inosine monophosphate and ammonia as part of purine nucleotide metabolism (3). Deficiency of MAD usually leads to mild symptoms, if any (4, 5), with some authors questioning its clinical significance (6). Other evidence, however, supports that a proportion of MAD-deficient patients are symptomatic (5, 7), with more recent studies demonstrating a deleterious impact on athletic performance (8, 9).

An association between MAD-deficiency and a variety of neuromuscular disorders has been noted, including (but not limited to) myositis, myasthenia gravis, myotonic dystrophy, neuropathies and other metabolic myopathies (1, 6, 10–14). Such cases are often considered to be a form of secondary or acquired MAD-deficiency. In contrast to the homozygous mutations seen in inherited cases, it has been speculated that acquired cases of MAD-deficiency may potentially arise through a “two hit” phenomenon. Here, an acquired neuromuscular disease may cause functionally significant MAD enzymatic deficiency in those with a predisposing heterozygous gene mutation (1). Conversely, Verjizl et al. found that all identified “secondary” cases in a Dutch population were in fact homozygous for the mutation, concluding that “secondary” cases represent merely a coincidental co-occurrence of inherited MAD deficiency with an unrelated neuromuscular disorder (6).

Idiopathic Inflammatory Myopathies (IIMs) are a distinct group of autoimmune conditions in which skeletal muscle inflammation leads to muscle weakness. Some IIM patients in whom muscle inflammation is suppressed with immunosuppressive/immunomodulatory treatment, as gauged by surrogate markers of muscle inflammation (reduced CK levels and T2 signal on muscle MRI), have persistently reduced strength. Other pathological processes within muscle aside from inflammation and necrosis have been postulated to contribute to ongoing weakness (15–18).

Three small studies have detected lower MAD activity in patients with IIM, though only a handful of cases were examined in these studies (1, 10, 11). In six patients with polymyositis (as diagnosed in 1991), reduced MAD activity was seen compared with controls. The reduction in MAD activity was found to correlate with the degree of myofibre necrosis, though it is unclear whether this was simply a reflection of necrotic cells or an inherent part of the underlying disease process (11). More recently, in a mouse model of myositis, it was noted that mice showing the disease phenotype had lower levels of MAD enzyme activity compared with healthy controls (19). Such literature suggests further investigation into a possible association between MAD and inflammatory myopathies is needed.

By using a much larger sample of inflammatory myopathy cases than have previously been examined, in this study we sought to (1) determine the proportion of IIM cases that are deficient in MAD and conversely, (2) determine the proportion of MAD-deficient cases which show features of muscle inflammation.

## Method

### Study design

We utilised two complementary approaches to investigate a possible relationship between MAD and IIM.

Firstly, we retrospectively reviewed muscle biopsy reports performed through the department of Anatomical Pathology, SA Pathology over a 17-year period between 30/6/2004 and 30/6/2021. MAD deficient cases over this period were identified through a keyword search of the laboratory’s electronic database of all biopsy reports.

The biopsy reports of all confirmed MAD-deficient cases were reviewed to identify any histological diagnoses as well as for features compatible with myositis, namely upregulation of MHC1/2, endomysial lymphocytic infiltration, and high CD45 or CD68 expression. Clinical details were sought for all MAD-deficient cases by reviewing the clinical information provided on biopsy request, as well as by searching the electronic health record systems for South Australian public hospitals. The following details were recorded: clinical diagnoses of neuromuscular disorders, presence of myalgia or weakness, serum levels of creatine kinase (CK), vitamin D, TSH, presence of antinuclear antibodies (ANA), antibodies to extractable nuclear antigens (ENA), myositis-specific and myositis-associated antibodies (including SRP, Mi-2A, Mi-2B, Tif1-Gamma, MDA5, NXP2, SAE1, KU, PM Scl, Jo-1, Pl-7, PL-12, EJ, OJ, Ro-52, SSA, SSB, Sm, RNP, Topoisomerase and anti-HMGCR).

For the second arm of the study, we prospectively stained for MAD-deficiency in all biopsy specimens analysed at SA Pathology (regardless of indication) for a 12-month period beginning 28/11/2020.

To identify IIM cases within both arms of the study, we searched the South Australian Myositis Database, which holds records of all adult muscle biopsies with a histological diagnosis of IIM made subsequent to 1980 through SA Pathology. All muscle biopsies within South Australia from public and private sectors are reported in this laboratory, and referrals are also received from Newcastle Hospital, New South Wales, and Darwin, Northern Territory. The laboratory uses well-validated criteria for diagnosis of Polymyositis, Dermatomyositis, Inclusion Body Myositis and Necrotising Myopathy, and biopsies are subject to peer review (20).

Testing for deficiency of MAD was performed using the original muscle biopsy enzyme-histochemical staining technique outlined by Fishbein (21). In brief, p-nitro blue tetrazolium chloride (NBT) and adenosine monophosphate (AMP) are combined with potassium chloride, titrated to a pH of 6.1 with sodium hydroxide. Dithiothreitol is added, and duplicate 9μm skeletal muscle frozen sections are added to the solution as well as to a negative control solution (which does not contain AMP). These are then incubated concurrently at room temperature. If MAD is present in the specimen, its activity on AMP results in ammonia production, raising the pH of the solution. This loss of acidity facilitates the reduction of NBT by the added thiol, producing deep blue colouration throughout the section. If the enzyme is absent from the specimen, the solution remains sufficiently acidic that NBT is not reduced, resulting in the absence of visible staining.

### Statistical analysis

This study was predominantly descriptive in nature. Univariate statistical analysis for relevant comparative data was performed using chi-square tests for categorical variables.

### Ethics approval

The study was approved by the Human Research Ethics Committee of the Royal Adelaide Hospital. Requirement for patient consent was waived based on the NHMRC statement on ethical research.

## Results

In the retrospective arm, 2,394 muscle biopsies were evaluated at SA pathology during the 17-year period between 30/6/2004–30/6/2021. Of these, 510 (21.3%) had a histological diagnosis of IIM. Over the same period, 30 confirmed cases of MAD deficiency were identified, yielding a rate of MAD-deficiency of at least 1.3%.

Of the 30 MAD-deficient cases identified, 26.7% (8/30) had a concurrent histological diagnosis of IIM, compared with 21.3% (510/2394) of all muscle biopsies conducted over the same period (*p* = 0.47). Of the 8 cases with dual IIM and MAD-deficiency (Table 1), 5 had necrotising myopathy, 1 had dermatomyositis and 2 had myositis-not-otherwise-specified. A possible 9th case of IIM with MAD-deficiency was identified from clinical notes (discharge diagnosis “autoimmune myositis”), though the histological diagnosis, and recorded peak CK of 159,000 U/L, was more suggestive of rhabdomyolysis.

**TABLE 1:** Characteristics of myoadenylate deaminase (MAD)-deficient cases with myositis.

	Year of diagnosis	Histological diagnosis other than MAD-deficiency	Documented clinical diagnosis	Peak CK (U/L)
1	2015	Necrotising myopathy	N/A	20,000
2	2015	Mild inflammatory myopathy (most in keeping with polymyositis/overlap myositis)	Inflammatory myopathy unspecified; Sjogren’s syndrome	149
3	2015	Inflammatory myopathy with overlapping features of necrotising myopathy	N/A	5,636
4	2016	Necrotising myopathy	N/A	4,000
5	2016	Necrotising myopathy	N/A	5,100
6	2018	Necrotising myopathy	Necrotising myopathy (HMGCR positive)	8,000
7	2020	Suggestive of dermatomyositis	Dermatomyositis (MDA5 positive); skin psoriasis treated with Risankizumab	22
8	2020	Necrotising myopathy; possible denervation	Myositis not otherwise specified (HMGCR positive)	867

CK, creatine kinase; MAD, myoadenylate deaminase; HMGCR, 3-hydroxy-3-methylglutaryl-CoA reductase.

Of the remaining 21 MAD-deficient cases (Table 2), identified neuromuscular diagnoses included muscular dystrophy (*n* = 1), alcohol-related myopathy (*n* = 1), viral myopathy (1), multiple sclerosis with spinal stenosis (1), spinal stenosis alone (1), Parkinson’s disease (1) motor neuron disease (1) and myofibrillar myopathy (1). The remaining 13 cases had no specific diagnosis.

**TABLE 2:** Characteristics of myoadenylate deaminase-deficient cases without myositis.

	Year of diagnosis	Histological diagnosis other than MAD-deficiency	Documented clinical diagnosis	Peak CK (U/L)
1	2005	Normal muscle	Undefined neuromuscular disorder	1,000
2	2006	Possible muscular dystrophy	Muscular dystrophy (possible limb girdle, sporadic)	600
3	2007	Minor non-specific myopathic abnormalities	N/A	7,500
4	2007	Rhabdomyolysis (Differential diagnosis polymyositis)	Rhabdomyolysis; “autoimmune myositis” (minimal documentation beyond this label)	158,000
5	2007	Normal muscle	Alcohol related myopathy	40
6	2007	Normal muscle	Primary MAD deficiency	66
7	2007	Normal muscle	N/A	153
8	2007	Normal muscle	N/A	875
9	2009	Mild (non-specific) atrophic myopathic changes	Suspected genetic disorder (undefined)	721
10	2009	Normal muscle	Viral myopathy	3,000
11	2010	Normal muscle	N/A	N/A
12	2011	Denervation	Spinal stenosis	86
13	2012	Features of chronic (subclinical) denervation, occasional necrotic and regenerative fibres of uncertain significance	Parkinsons disease	658
14	2013	Chronic denervation	Multiple sclerosis, spinal stenosis	345
15	2014	Hydroxychloroquine exposure (curvilinear bodies). Possible central core disease	Rheumatoid arthritis	637
16	2014	Normal muscle	Primary MAD deficiency	8,627
17	2015	Mild non-specific myopathic features	N/A	1,984
18	2015	Myofibrillar myopathy (eg myotilinopathy or filaminopathy)	N/A	71
19	2016	Normal muscle	N/A	154
20	2017	Non-specific myopathic changes without inflammation or necrosis	Primary MAD deficiency; Rheumatoid arthritis	346
21	2017	Denervation	Motor neuron disease	1,853
22	2020	Non-specific moderate myopathic features with scattered necrotic fibres. Some COX negative fibres (can raise possibility of a mitochondrial myopathy)	Undefined myopathy	937

CK, creatine kinase; MAD, myoadenylate deaminase.

Amongst MAD-deficient cases, CK was elevated in 20/29 patients in whom CK values were available (see Supplementary Tables 1 and 2), with median peak CK of 867 U/L (normal range <150 U/L). At least 50% of patients (15/30) described myalgia and 73% (22/30) described weakness. Only 3/30 cases had a documented history of statin use.

Serological testing in MAD-deficient cases, where available, showed antinuclear antibodies in 3/16, myositis-specific-antibodies in 1/9 (anti-MDA-5), anti-HMGCR in 2/6, and 0/16 cases had antibodies to extractable nuclear antigens.

TSH levels were within reference range for all 18 cases in whom this test was available, with mild vitamin D deficiency in 4/11 patients (lowest 43 nmol/L).

Histology reports of MAD-deficient cases were examined for features of inflammation. We observed increased MHC1 expression in 17% (5/30), increased MHC2 expression in 6.7% (2/30), lymphocytic infiltrates in 10% (3/30), CD45 expression in 17% (5/30) and CD68 expression in 13% (4/30). As could be expected, inflammatory features on biopsy were more commonly seen in the 8 cases with a concurrent diagnosis of myositis (see Supplementary Table 1) compared to those without myositis (Supplementary Table 2).

In the prospective arm, there were no detected cases of MAD-deficiency (0/173), despite a total of 39 cases being diagnosed with IIM (22.5%) in this 12-month period. Contemporary re-testing of previously identified MAD-deficient samples from earlier years demonstrated that the staining technique employed in 2021 does identify MAD-deficiency when present.

## Discussion

Our results show that IIM and MAD deficiency, as detected by enzyme histochemical staining, are not strongly associated, with no significant difference in the rates of myositis diagnosis between muscle biopsy samples with and without MAD-deficiency (26.7% vs 21.3%, *P* = 0.47). Furthermore, in the prospective arm, we identified no MAD-deficient cases among the 39 IIM cases detected in the latest 12-month period.

Clinical features of myalgia, weakness and raised CK were common in MAD-deficient patients, though this is not unexpected given that all patients in the study underwent muscle biopsy based on clinical indication. Inflammatory features on MAD-deficient biopsy specimens were not prominent, especially considering that more than 20% of all biopsy samples examined by our laboratory during the study period had a histological diagnosis of myositis.

We found a slightly lower than expected rate of MAD-deficiency, 0% in the prospective arm (*n* = 173), and 1.3% in the retrospective arm, though the latter may have been higher had staining been conducted on all samples during that period. In comparison to previously published rates of MAD-deficiency in the general population, a rate of 1.8% was observed in the largest muscle biopsy series to date in Holland (6). Previous studies of American, German and Dutch populations have found allele frequencies predicting homozygosity (and hence functional deficiency) in 1–3% of subjects, though notably zero mutant alleles were detected among 106 Japanese patients (2, 3, 5). South Australia has a multicultural population (though predominantly Caucasian), and ethnic diversity may account for our relatively lower rates of MAD-deficiency.

One potential limitation of the study is that the staining technique used for detecting MAD activity, while effective and routinely used in clinical practice, could theoretically be less sensitive in detecting subtle partial enzyme deficiencies. Alternative measures of detecting MAD activity have been devised, but are not readily available nor commonly used in clinical practice, and would be impractical for investigating our large number of myositis cases (11). The clinical significance of such mild enzyme deficiency (if present) is also questionable, considering that even complete enzymatic deficiency does not consistently lead to muscle symptoms (5, 6). Our study is unable to exclude very mild reductions in MAD activity in myositis patients, but has shown that significant degrees of deficiency are not more commonly seen in the myositis patient population.

Genetic testing was not performed on any of our patients, however, we would not expect any patients with the homozygous mutation to be missed by the staining technique. We recognise that secondary cases have been hypothesised by some to arise in patients heterozygous for the mutation (1), though this is not without dispute (6). As others have pointed out, given the high population prevalence of heterozygosity for the C34T mutation (18%), it is hard to attribute symptoms to acquired MAD deficiency (over and above the known neuromuscular disorder) without at least some proven measure that enzyme activity is reduced (6). We thus believe that clinically relevant degrees of enzyme deficiency are unlikely to be at play if not detectable on our assay assessing *in vitro* function of the enzyme.

Other limitations of our study include the incomplete clinical records for some patients (particularly those referred for biopsy assessment from interstate) and the small numbers in the prospective arm. In the retrospective arm of the study, with larger numbers, we acknowledge that international diagnostic criteria for IIM have changed over the 17-year study duration, and consequently classification may not be perfectly homogenous.

Despite these limitations, the results of our study show that inflammatory myopathies and MAD deficiency, as detectable by enzyme histochemical staining, do not appear to be strongly associated. Secondary MAD-deficiency is thus unlikely to be a major driver of symptoms in myositis patients. Uncertainty still remains around whether other non-inflammatory mechanisms may contribute to weakness in these conditions. Alternative proposed mechanisms include endoplasmic reticulum stress and reactive oxygen species, and further research into the role of other such pathways may prove fruitful in the search to better understand the pathobiology of inflammatory myopathies (18).

## Data availability statement

The raw data supporting the conclusions of this article will be made available by the authors, without undue reservation.

## Ethics statement

The studies involving human participants were reviewed and approved by the Human Research Ethics Committee of the Royal Adelaide Hospital. Written informed consent for participation was not required for this study in accordance with the national legislation and the institutional requirements.

## Author contributions

MW and VL conceived and designed the study, with input on technical aspects from KC. MW, KC, and BG collected data. MW analysed the data with input from VL. All authors helped shape the overall approach to the research and wrote the manuscript.

## Abbreviations

CK: creatine kinase
ENA: extractable nuclear antigens
IIMs: idiopathic inflammatory myopathies
MAD: myoadenylate deaminase
AMP: adenosine monophosphate
NBT: nitro blue tetrazolium.
