# Supplementary material for: Secondary myoadenylate deaminase deficiency is not a common feature of inflammatory myopathies: A descriptive study
Source: Front Med (Lausanne). 2022 Nov 23;9:1061722. doi: 10.3389/fmed.2022.1061722 (PMC9727292; doi:10.3389/fmed.2022.1061722)
Supplement: Supplementary file 1 [file Table_1.docx]

Supplementary Table 1

**Supplementary Table 1: Muscle Biopsy Features of MAD-Deficient Cases With Myositis**

| **Case No.** | **Histological Diagnosis other than MAD-deficiency** | **Documented Clinical Diagnosis** | **Raised CK (>250 U/L)** | **Peak CK (U/L)** | **MHC1 upregulation** | **MHC2 upregulation** | **Increased lymphocytes** | **High CD45 expression** | **High CD68 expression** | **Necrosis** | **Regeneration** |
| --- | --- | --- | --- | --- | --- | --- | --- | --- | --- | --- | --- |
| 1 | Necrotising myopathy | N/A | Y | 20,000 | N | N | N | N | N | Y | Y |
| 2 | Mild inflammatory myopathy (most in keeping with polymyositis/overlap myositis) | Inflammatory myopathy unspecified; Sjogren’s syndrome | N | 149 | Y | Y | Y | Y | Y | N | Y |
| 3 | Inflammatory myopathy with overlapping features of necrotising myopathy | N/A | Y | 5636 | N | N | N | Y | Y | Y | Y |
| 4 | Necrotising myopathy | N/A | Y | 4000 | N | N | N | N | N | Y | Y |
| 5 | Necrotising myopathy | N/A | Y | 5100 | Y | N | N | N | N | Y | Y |
| 6 | Necrotising myopathy | Necrotising myopathy **(HMGCR positive)** | Y | 8000 | N | N | N | N | N | Y | Y |
| 7 | Suggestive of dermatomyositis | Dermatomyositis **(MDA5 positive)**; skin psoriasis on Risankizumab | N | 22 | Y | N | Y | Y | Y | N | N |
| 8 | Necrotising myopathy; possible denervation | Myositis not otherwise specified **(HMGCR positive)** | Y | 867 | Y | Y | Y | Y | N | Y | Y |

Abbreviations: CK (Creatine Kinase), MAD (Myoadenylate Deaminase), HMGCR (3-hydroxy-3-methylglutaryl-CoA Reductase)
