# Supplementary material for: Secondary myoadenylate deaminase deficiency is not a common feature of inflammatory myopathies: A descriptive study
Source: Front Med (Lausanne). 2022 Nov 23;9:1061722. doi: 10.3389/fmed.2022.1061722 (PMC9727292; doi:10.3389/fmed.2022.1061722)
Supplement: Supplementary file 2 [file Table_2.docx]

Supplementary Table 2

**Supplementary Table 2: Muscle Biopsy Features of MAD-Deficient Cases Without Myositis**

| **Case No.** | **Histological Diagnosis other than MAD-deficiency** | **Documented Clinical Diagnosis** | **Raised CK (>250 U/L)** | **Peak CK (U/L)** | **MHC1 upregulation** | **MHC2 upregulation** | **Increased lymphocytes** | **High CD45 expression** | **High CD68 expression** | **Necrosis** | **Regeneration** |
| --- | --- | --- | --- | --- | --- | --- | --- | --- | --- | --- | --- |
| 1 | Normal muscle | Undefined neuromuscular disorder | Y | 1000 | N | N | N | N | N | N | N |
| 2 | Possible muscular dystrophy | Muscular dystrophy (possible limb girdle, sporadic) | Y | 600 | N | N | N | N | N | N | N |
| 3 | Minor nonspecific myopathic abnormalities | N/A | Y | 7500 | N | N | N | N | N | N | N |
| 4 | Rhabdomyolysis (Differential diagnosis polymyositis) | Rhabdomyolysis; "autoimmune myositis" (minimal documentation beyond this label) | Y | 158,000 | N | N | N | N | N | Y | Y |
| 5 | Normal muscle | Alcohol related myopathy | N | 40 | N | N | N | N | N | N | N |
| 6 | Normal muscle | Primary MAD deficiency | N | 66 | N | N | N | N | N | N | N |
| 7 | Normal muscle | N/A | N | 153 | N | N | N | N | N | N | N |
| 8 | Normal muscle | N/A | Y | 875 | N | N | N | N | N | N | N |
| 9 | Mild (non-specific) atrophic myopathic changes | Suspected genetic disorder (undefined) | Y | 721 | N | N | N | N | N | N | N |
| 10 | Normal muscle | Viral myopathy | Y | 3000 | N | N | N | N | N | N | N |
| 11 | Normal muscle | N/A | N/A | N/A | N | N | N | N | N | N | N |
| 12 | Denervation | Spinal stenosis | N | 86 | Y | N | N | N | N | N | N |
| 13 | Features of chronic (subclinical) denervation, occasional necrotic and regenerative fibres of uncertain significance | Parkinsons disease | Y | 658 | N | N | N | N | N | Y | Y |
| 14 | Chronic denervation | Multiple sclerosis, spinal stenosis | Y | 345 | N | N | N | N | N | N | N |
| 15 | Hydroxychloroquine exposure (curvilinear bodies). Possible central core disease | Rheumatoid arthritis | Y | 637 | N | N | N | N | N | N | N |
| 16 | Normal muscle | Primary MAD deficiency | Y | 8627 | N | N | N | N | N | N | N |
| 17 | Mild non-specific myopathic features | N/A | Y | 1984 | N | N | N | N | N | N | Y |
| 18 | Myofibrillar myopathy (eg myotilinopathy or filaminopathy) | N/A | N | 71 | N | N | N | N | N | N | N |
| 19 | Normal muscle | N/A | N | 154 | N | N | N | N | N | N | N |
| 20 | Non-specific myopathic changes without inflammation or necrosis | Primary MAD deficiency; Rheumatoid arthritis | Y | 346 | N | N | N | N | N | N | N |
| 21 | Denervation | Motor neuron disease | Y | 1853 | N | N | N | N | N | N | N |
| 22 | Non-specific moderate myopathic features with scattered necrotic fibres. Some COX negative fibres (can raise possibility of a mitochondrial myopathy) | Undefined myopathy | Y | 937 | N | N | N | Y | Y | Y | N |

Abbreviations: CK (Creatine Kinase), MAD (Myoadenylate Deaminase)
